# Extrusion – back to the future: Using an established technique to reform automated chemical synthesis

**DOI:** 10.3762/bjoc.13.9

**Published:** 2017-01-11

**Authors:** Deborah E Crawford

**Affiliations:** 1 Queen’s University Belfast, School of Chemistry and Chemical Engineering, David Keir Building, 39–123 Stranmillis Road, Belfast, BT9 5AG, Northern Ireland, UK

**Keywords:** continuous, extrusion, industry, organic, synthesis

## Abstract

Herein, the benefits which extrusion can provide for the automated continuous synthesis of organic compounds are highlighted. Extrusion is a well-established technique that has a vital role in the manufacturing processes of polymers, pharmaceuticals and food products. Furthermore, this technique has recently been applied to the solvent-free continuous synthesis of co-crystals and coordination compounds including metal-organic frameworks (MOFs). To date, a vast amount of research has already been conducted into reactive extrusion (REX), particularly in the polymer industry, which in many cases has involved organic transformations, however, it has not received significant recognition for this. This review highlights these transformations and discusses how this previous research can be applied to the future of organic compound manufacture.

## Review

### Extrusion methodology

**Figure 1:**
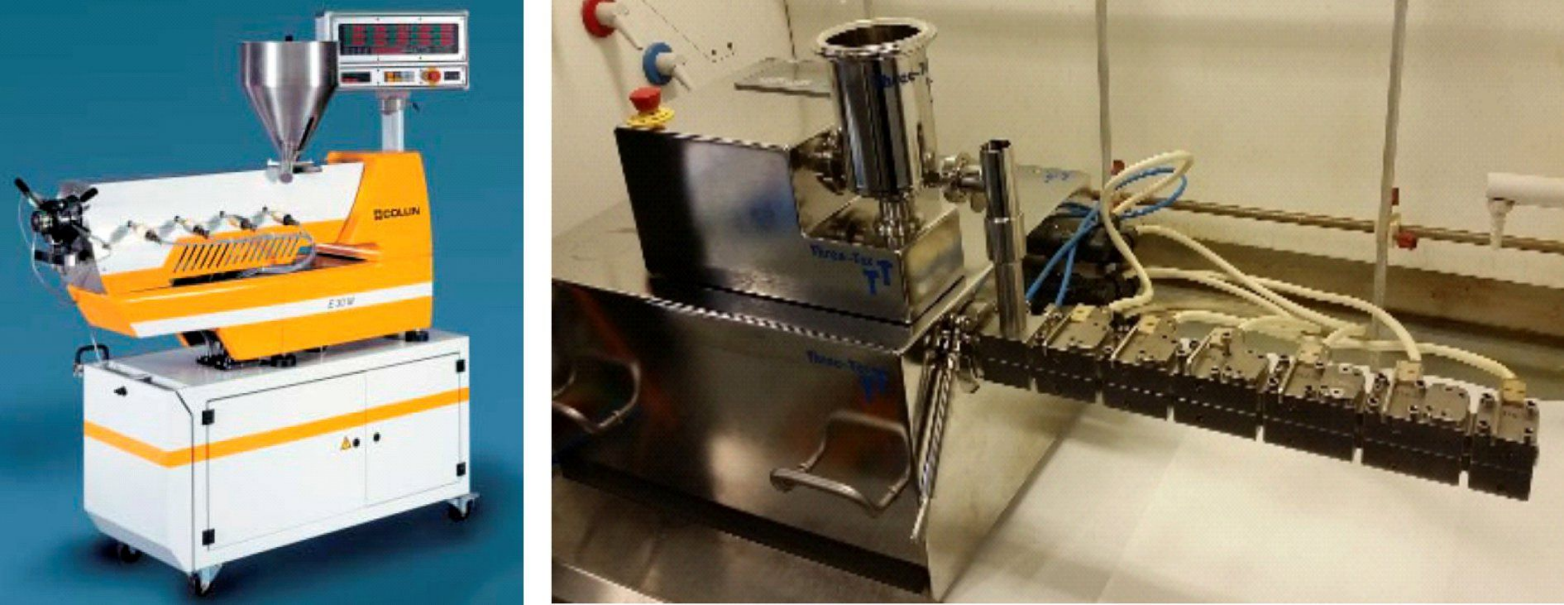
Typical pilot scale single screw extruder (left) and a laboratory scale twin screw extruder (right).

Extrusion is an umbrella term covering a family of processes that involves the movement of material through a confined space, most typically along a set of screws – screw extrusion. There are two main types of screw extrusion – single (SSE) and twin screw (TSE) (Figure 1) [1–3]. As the names suggest, SSE involves the movement of material by one screw, whereas TSE, which is more frequently employed, involves the movement of material by two, i.e., the material is conveyed from one screw to the other as it makes its way along the extruder barrel [4].

**Figure 2:**
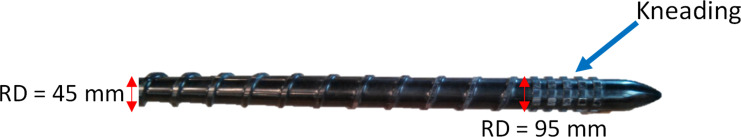
PTFE screw employed in single screw extrusion, with increasing root diameter (RD) from 45 mm to 95 mm and a final kneading section.

Both techniques process materials by mixing, heating and also by applying mechanical energy. The main forces present in an extrusion process are compression forces and shear. However, the methodology of each technique differs significantly, as well as the applications for which they are employed, for example SSE is typically used to carry out hot melt extrusion (HME), where the emphasis is on the melting of material for thorough mixing and processing [5]. The process can be adapted via modulation of the screw, as depicted in Figure 2, to make the process more efficient. An industrial single screw extruder typically has a screw diameter ranging from anywhere between 1 inch and 24 inches. Principally, the root diameter (the diameter of the central part of the screw) of the screw increases along its length, this is to i) provide greater free volume at the beginning of the ‘starve-fed’ extruder for maximum feeding of material and ii) to increase the compressive forces at a later stage of the process, as a result of the volume being reduced, whilst a large amount of material is still present [3]. This also results in an increase of the shear applied to the material, as it experiences friction from moving between both the screw and the barrel walls. In addition, a kneading segment can be added at the end of the screw, to provide a region of intense mixing (with increased shear) before the material exists the extruder. It must be noted that the flow of material along a single screw extruder is essentially reliant on the feeding of material into the barrel, which provides a forward pressure so that the material can exit the barrel [5].

**Figure 3:**
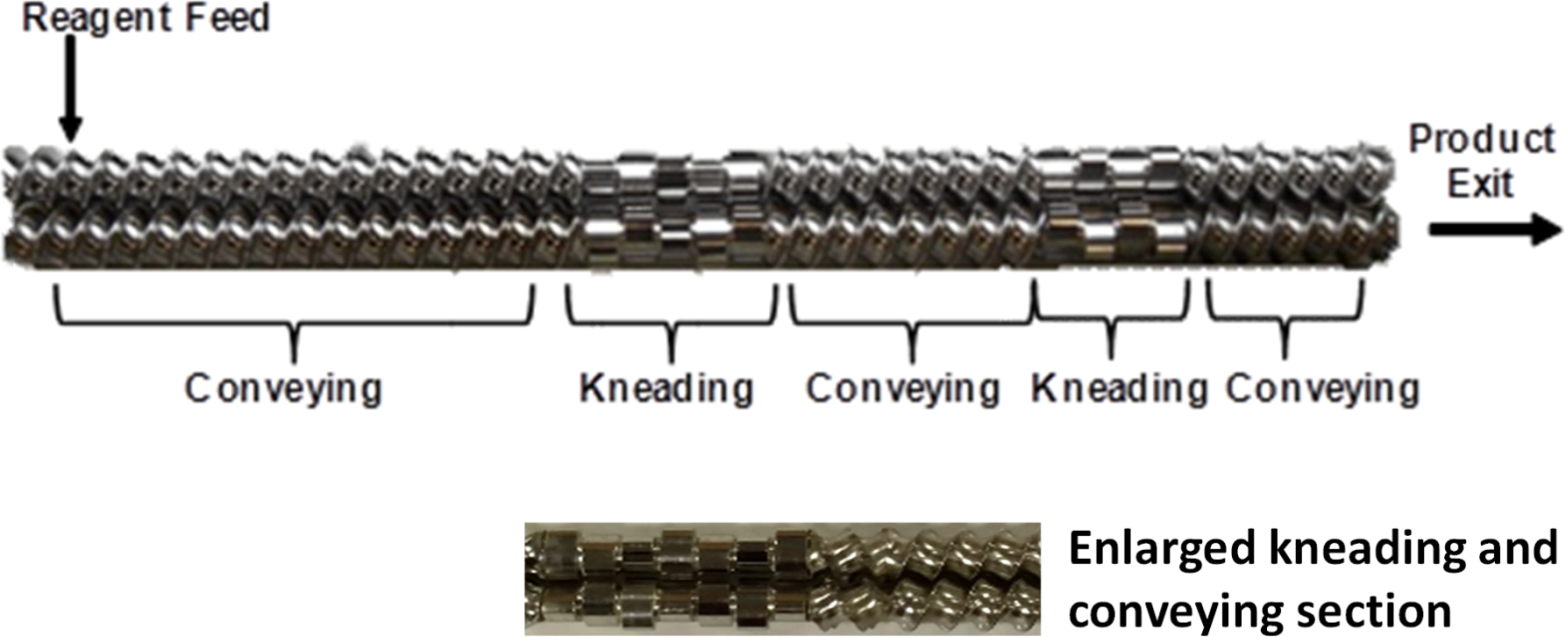
Modulated stainless steel intermeshing co-rotating screws employed typically in twin screw extrusion, comprised of conveying and kneading segments.

TSE however employs two intermeshing screws and it is mainly the movement of material from one screw to the other, and back again, that conveys the material along the barrel. The configuration of these screws is generally more intricate and typically comprised of a series of alternating conveying and kneading segments (Figure 3). The main advantage of employing modulated screws is that the screw configuration can be adjusted for each process. The conveying segments are generally of quite large channel depth, i.e., the radial distance between the flight tip and the screw root (ca. 2–3 mm for smaller extruders, and several centimetres or inches for extruders employed in industry), but again as with SSE, this channel depth decreases along the screw length, resulting in an increase of the compressive forces and shear. The equivalent to channel depth within continuous flow chemistry is typically very narrow of several millimetres. Furthermore, the kneading segments can be positioned at angles of 30^o^, 60^o^ and 90^o^ relative to each other, with the latter angle providing the greatest kneading (and shear). The kneading section can be quite hostile as it involves not just mixing, but also the grinding of the material, which resultantly leads to changes in the material properties, most commonly its rheology [3]. Furthermore, the mechanical energy applied to the system can be controlled by the screw profile, as well as the residence time which is not only dependent on screw speed, but on the configuration too, allowing it to be prolonged if required. Modification of the screw profile, by inserting additional segments or those of a different configuration, e.g., toothed segments, can result in some control of the temperature as well, screw segments that apply greater mechanical energy may result in a greater amount of frictional heat being produced, particularly in comparison to those segments which provide less vigorous kneading.

Both single and twin screw extruders range from 10–443 mm in screw diameter [6] and extrusion processes are scalable to produce large quantities of materials in the range of tonnes per hour as a result of the extensive engineering research. Herein, a focused discussion of the reactive processes carried out by extrusion is provided. A substantial amount of the organic transformations carried out by extrusion has been in the polymer industry, however, most of these processes have been overlooked by synthetic chemists. In fact, the authors of these REX processes have focused mainly on optimising the process conditions and have not discussed the chemistry itself. It is hoped that this article will show readers that there is an extensive amount of research into continuous organic transformations by extrusion and encourage them to consider the potential that extrusion holds for continuous chemical synthesis, particularly under solvent-free conditions.

### Reactive extrusion (REX)

Extrusion is employed most frequently in the polymer industry, generally for the dispersion of materials (e.g., graphene or quantum dots) into polymers [7]. However, REX is also employed as a technique to synthesise polymers or to carry out post synthetic polymer modification (e.g., functionalisation of polymer chains) via organic transformations, which in turn alters the properties of the materials [6].

Initially, the polymer industry employed only batch mixers to synthesise polymers and carry out post synthetic modification (PSM), however, this proved difficult and inefficient. This was due to a dramatic increase or change in the viscosity and rheology of the material, a common feature of REX, and therefore as reactions proceed, they can become very difficult to mix efficiently, leading to low conversions. A second problem associated with the change in viscosity is the resultant poor heat transfer, meaning that longer heating times are required, which often leads to polymer degradation. Employing extrusion overcame these issues. REX is now initially carried out in a batch mixer and the material is subsequently transferred to an extruder. This allows for fresh, thin reactive surfaces to be exposed, which encourages these reactions to go to completion [8]. Overall, the time required to carry out these processes was reduced, as well as the time during which the material is exposed to heat, therefore preventing polymer degradation [9–10].

There are five main types of reactive polymerisation for which extrusion has been employed, containing some clear examples of organic transformations. One of the most common forms is bulk polymerisation, involving the formation of a polymer (linear, branched and crosslinked) of high molecular weight starting from a series of monomers [11]. Bulk polymerisation will be discussed in detail, however, it is worthwhile noting the other various reaction types explored by extrusion:

- Grafting reactions – a grafted polymer is synthesised from the reaction of a polymer and a functionalised monomer [12].
- Functionalisation – this takes place on already prepared polymers, when the polymer is either functionalised by the modification or addition of a functional group [13].
- Controlled degradation – degradation and crosslinking of polymers to produce a product with controlled molecular weight distribution. This results in a higher number of active sites that can later be used for grafting [13].
- Reactive blending – this involves the extrusion of two or more compatible polymer blends, leading to the formation of a polymer–polymer complex [13].

Bulk polymerisation involves several common organic transformations, including living polymerisation, polyaddition, radical and polycondensation polymerisation. Living polymerisation, the most common transformation, involves the constant growth of a polymer chain where the ability to terminate the reaction is removed [14]. Again, there are several types of transformation including ionic polymerisation, ring opening metathesis, free radical and growth polycondensations [15]. All of which have been shown to be successful by extrusion.

#### Living polymerisation

**Scheme 1:**
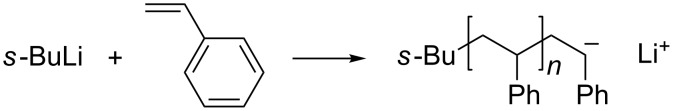
Polymerisation of styrene using *s*-BuLi as an initiator.

There have been several publications on the use of living anionic polymerisations in the preparation of polystyrene. Höcker et al. have investigated the role of extrusion in i) the preparation of this polymer and ii) the sequential postsynthetic modification of polystyrene by isoprene [16]. The authors focus mainly on the engineering aspects of the project rather than the chemical reaction itself. The first part of the process involves the chemical reaction between styrene and *s*-BuLi, which is employed as an initiator (Scheme 1). The *s*-BuLi reacts with the double bond of styrene, initiating a homopolymerisation process. Once all the monomer is consumed, the polymer has a stable anionic polymer chain, which allows for further functionalisation by reaction with electrophilic functional groups.

The solution-based living polymerisation reactions are typically dependent on the solvent employed, temperature and concentration [17], however, when they are conducted by extrusion, they are carried out under solvent-free conditions, which is a major advantage. It must also be noted that to carry out a reaction solvent-free, when a pyrophoric reagent, such as *s*-BuLi, is involved is quite remarkable, and it is carried out as a continuous process and not on a small scale. This suggests that solvent-free extrusion can be on par with continuous flow technology, allowing a wider range of hazardous reagents to be used continuously and on large scale.

**Scheme 2:**
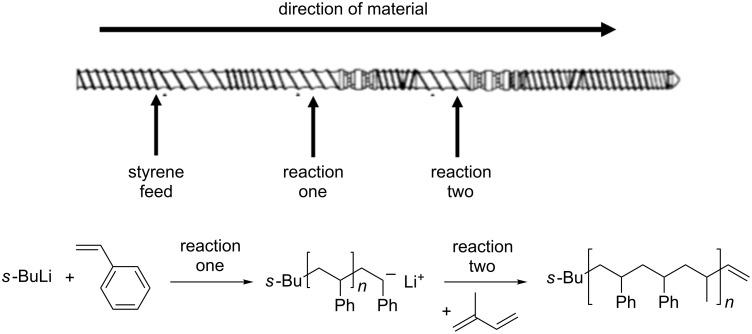
Telescoping process of the formation of polystyrene, followed by post polymerisation functionalisation with isoprene. The figure depicts the screw configuration employed and each reaction. Adapted from [16].

Furthermore, Höcker et al. report the post polymerisation of polystyrene with isoprene, which is actually carried out in the same processing line as the polymerisation of styrene, i.e., styrene is polymerised initially in the extruder barrel and isoprene is fed into the barrel at a later point to react with polystyrene in a second reaction (Scheme 2) [16]. This is an example of telescoping which is considered to be very advantageous in continuous flow technology for example. As a result of being able to carry out polymerisation and post polymerisation functionalisation by TSE, different polymer geometries can be achieved, for example a star or comb-shaped polymer [18–19]. This indicates that large molecules of well-defined architecture, in addition to polymers, could be synthesised by TSE. It must also be noted that these processes were optimised in order to have throughput rates of ca. 3–10 kg h^−1^ (after both transformations have been carried out) [16]. Unfortunately, there is no example in the literature for this reaction carried out in batch, however, Meyer reports on the general scalability of batch polymerisation and comments that up to 200 kg d^−1^ quantities can be obtained. It must be noted, however, that the reaction reported by Höcker involves the use of *s*-BuLi, making the process more difficult, but still a greater throughput rate is obtained than that predicted by Meyer [20].

Free radical polymerisation involving deactivation polymerisation, iniferter polymerisation and reversible addition fragmentation chain transfer (RAFT) polymerisation, amongst others [21], has been studied extensively by extrusion to produce, for example, branched polypropylene, polyethylene and polylactide polymers. The process involves the production of a free radical at the end of an active polymer chain, and is further characterised as living free radical polymerisation due to the complete absence of a termination reaction [22]. Narayan et al. report the branching of polylactide by TSE, during which the molecular weight of polylactide was increased dramatically at 170–180 °C [23]. This work highlights another advantage of extrusion in that the barrel can have separate heating zones (some also provide cooling), allowing the temperatures to be varied along the production line. In addition, due to the low free volume of the extruder barrel, but the high throughput rates achievable by extrusion, the material has a resultantly higher surface area exposed directly to heat. The material is exposed to heat usually only for a couple of minutes, which then avoids polymer degradation [3].

**Scheme 3:**
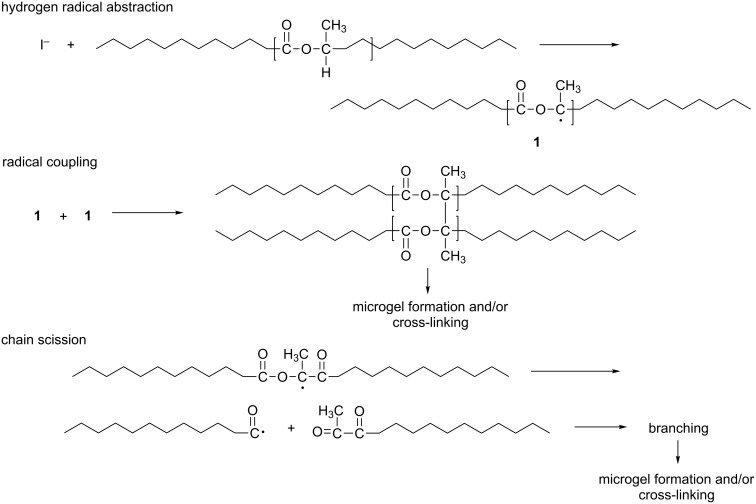
Proposed mechanism for the branching of polylactide. Adapted from [23].

Narayan reports the addition of an initiator, Lupersol, a di-tertiary alkyl peroxide which produces free radicals in bulk, to the formation of polylactide. Another advantage is that Lupersol is a food additive and is approved by the Food and Drug Administration (FDA). Furthermore, the authors hypothesise a mechanism by which the branching of polylactide is occurring, suggesting that the initial polymer undergoes a hydrogen radical abstraction, followed by radical coupling and finally chain scission (Scheme 3) [23].

#### Polyaddition polymerisation

**Scheme 4:**
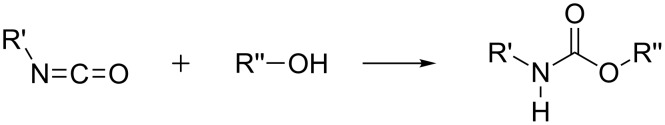
Chemical reaction between isocyanate and an alcohol to form polyurethane.

**Figure 4:**
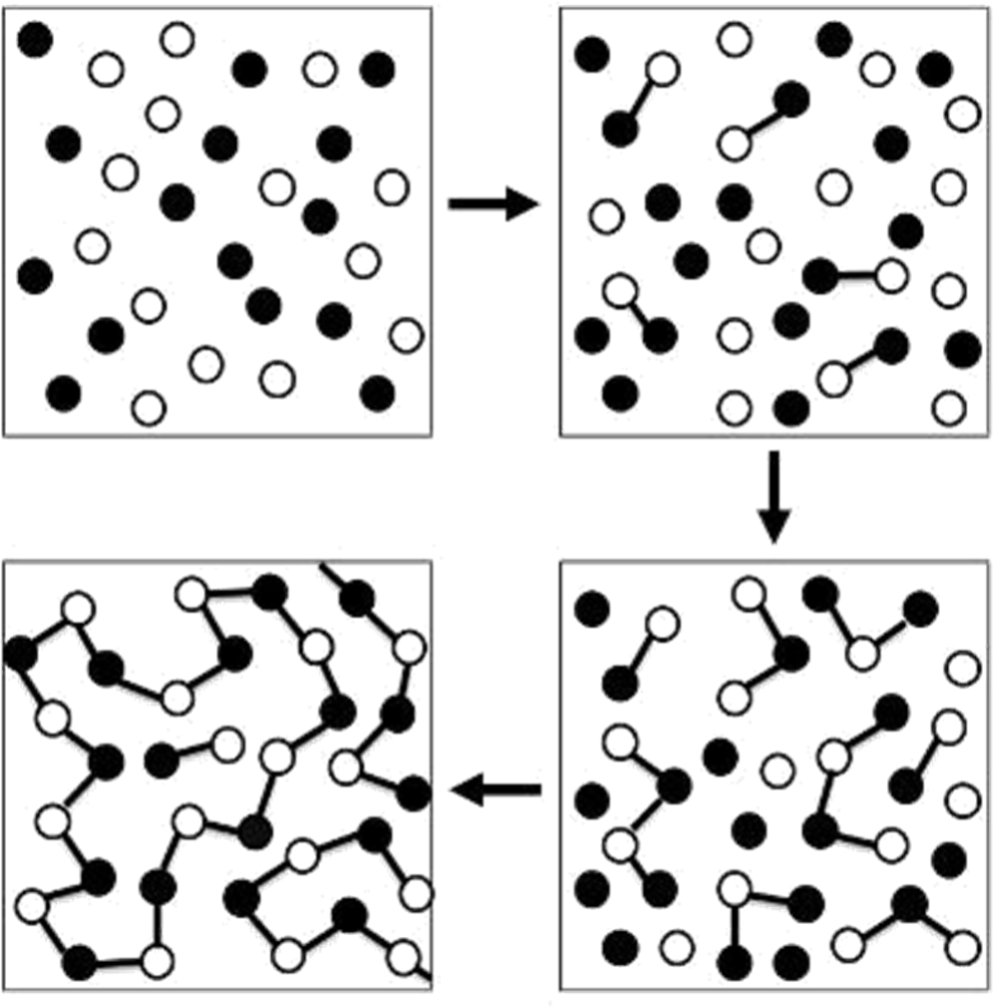
Representative diagram explaining the process involved in step growth polymerisation, which involves the formation of a dimer, then trimer followed by oligomer synthesis. Taken from [24].

Another common form of polymerisation is polyaddition polymerisation, an example is the formation of polyurethane from a reaction between an isocyanate and a hydroxy functional group (Scheme 4). Polyaddition involves the addition of monomers onto an actively growing polymer chain; however, there is also ‘step growth polymerisation’ which is employed in the formation of polyurethane. Step growth polymerisation involves a gradual approach to the polymer by initially forming a dimer from multifunctional monomers, which then forms a trimer, then oligomer and finally a polymer (Figure 4) [24].

Kim and Hyun report the synthesis of polyurethane, discussing the associated numerical simulation they conducted to determine the dependency of shear rate on viscosity, rheology and the kinetics of formation also. The authors report that a reaction between 4,4’-diphenylmethane diisocyanate, polycaprolactonediol and 1,4-butanediol takes place in a twin screw extruder, employing a screw speed of 15 rpm and a temperature of 60 °C [25]. This transformation was conducted in the presence of a catalyst – dibutyltin diaurate. The authors focus on the processing of this reaction rather than the chemistry taking place itself, however, this is one of the most traditional organic transformations carried out by TSE to date, carried out on a continuous scale whilst being metal catalysed by an organotin compound. It must be highlighted that the residence times for these reactions are relatively short at ca. 10 minutes, particularly upon comparison with the time required to carry out conventional organic synthesis, yet the process still forms the desired polymers of high molecular weight at moderate temperatures of 60 °C [25].

#### Polycondensation polymerisation

**Scheme 5:**
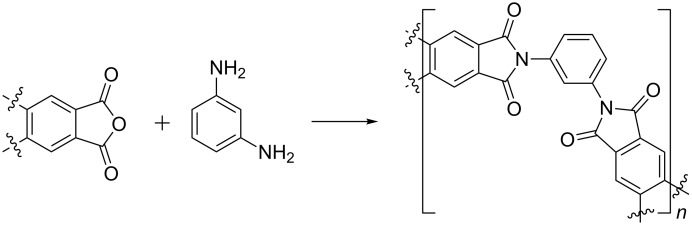
Generic polycondensation reaction to produce polyamides.

Finally, another important example of organic synthesis in the production of polymers is the polycondensation reaction to produce polymers such as polyamides. There are numerous patents on this application of polymer extrusion [26–28]. The reactions work very well by TSE as a result of being able to heat the extruder barrel to temperatures greater than that of boiling water. As a result, water (reaction byproduct) is removed during the extrusion process, driving the reactions to completion. Typically, reactions are carried out between diamines and an anhydride, dicarboxylic acid or a dicarbonyl compound (Scheme 5). Takekoshi et al. released a patent demonstrating the ability of extrusion to form a variety of different polyimides by extrusion in a completely solvent-free, continuous manner. Polycondensations were performed at temperatures between 210–350 °C, it can therefore be speculated that the high temperatures are required to accelerate the polymerisation reaction, rather than just drive off the water byproduct [29]. Batch synthesis of polyimide polymers typically involves mixing for 48 hours at temperatures ranging from room temperature to 250 °C [30].

### Chemical synthesis by extrusion

Extrusion is heavily relied upon within the pharmaceutical industry with regards to the formulation of drugs and their incorporation into drug delivery systems. However, extrusion has not been employed to carry out any organic compound or active pharmaceutical ingredient (API) synthesis in this industry. There has been some research in the last decade demonstrating the preparation of cocrystals by hot melt extrusion (HME) and liquid assisted extrusion. This work has been essentially conducted by Amgen, preparing cocrystals consisting of a pharmaceutical component [31–33]. There is currently extensive research being carried out into the effectiveness of cocrystals as medicinal products due to the higher dissolution rates they provide. Therefore, in order to employ cocrystals as drugs available to patients, not only is research into their bioavailability being conducted, but also into manufacturing techniques that could be utilised for their production. Currently, research into non-solvent based synthetic methods is being pursued to eliminate the influence that the solvent has over the cocrystal formed.

#### Cocrystal formation

In 2009, Alvarez-Nunez et al. of Amgen used TSE to scale up the synthesis of a cocrystal which had already been reported to be synthesised successfully by ball milling (employing liquid-assisted grinding (LAG)). This was the first example demonstrating that mechanochemical synthesis could be scaled up to several hundred grams and carried out continuously by employing hot melt extrusion (HME) [31,34]. Initially two cocrystals were optimised – a cocrystal formed from caffeine and oxalic acid and another consisting of AMG517 (a selective TRPV1 antagonist) and sorbic acid [31].

Since the publication of this work, Moradiya et al. (of Amgen) have reported the synthesis of carbamazepine-saccharin cocrystals by both TSE and SSE techniques [35]. Moradiya et al. found some difficulties in regards to maintaining an exact stoichiometry of cocrystal components – any deviation from the correct stoichiometry could potentially lead to undesirable variations in the properties of the product. Kulkarni et al., however, demonstrated that this issue could be resolved by careful manipulation of the extruder temperature [36], demonstrating that in the extrusion of a 2:1 mixture of caffeine/malic acid, extrusion temperatures of below 104 °C favoured the formation of a 1:1 product. Increasing to above 104 °C however resulted in the subsequent melting of the 1:1 product, followed by formation of the desired 2:1 cocrystal.

There are now several examples of cocrystal formation by HME present in the literature (<30 publications, mainly from researchers at Amgen) and studies on the extrusion process itself with regards to cocrystal manufacture is also gaining momentum. There are a few publications investigating the effect of screw speed and temperature on the process [37]. There is also an example in the literature demonstrating the utilisation of near-infrared spectroscopy for online monitoring to determine where in the extruder the cocrystal begins to form. Consequently this also provides feedback regarding screw configuration and deductions can be made as to whether sufficient mechanical energy is being applied in order to achieve 100% conversion to product for example [38].

Mechanistically, it was initially believed that the formation of a eutectic was vital to the formation of a cocrystal, but it has been reported that this is not always the case, in some cases it is the effect of high temperatures and screw configurations that has had the greatest influence. Furthermore, extrusion not only provides advantages to the formation of cocrystals by improving the manufacturing process, it has also been demonstrated to improve the properties of the materials. Alvarez-Nunez et al. report that in the formation of AMG517-sorbic acid cocrystal by extrusion, the N_2_ Brunauer–Emmett–Teller (BET) surface area was greater than the conventionally prepared cocrystals, and there were also improvements to the bulk density and flow properties of the material [31]. As a result of these superior material properties, a final milling process step typically employed in the conventional synthesis to increase the surface area was removed.

#### Deep eutectic solvents

Deep eutectic solvents (DESs) – regarded as a new generation of ionic liquids – are two-component ionic solvents with melting points lower than either constituent of the mixture [39–42]. These materials are receiving a lot of attention due to their potential applications in metal deposition and as green media in chemical reactions [43]. James et al. have reported the preparation of DESs Reline 200 (choline chloride:urea, 1:2), choline chloride:zinc chloride (1:2) and choline chloride:D-fructose (1.6:1) by TSE [44]. Typically they are prepared by batch heating, but this is not always very effective on large scale, especially as processing of these mixtures results in a dramatic increase in viscosity, this then results in an uneven distribution of each component in the mixture [45]. Furthermore, it was reported that batch heating also resulted in the thermal degradation of choline chloride:D-fructose DES due to the caramelisation of D-fructose [45].

**Figure 5:**
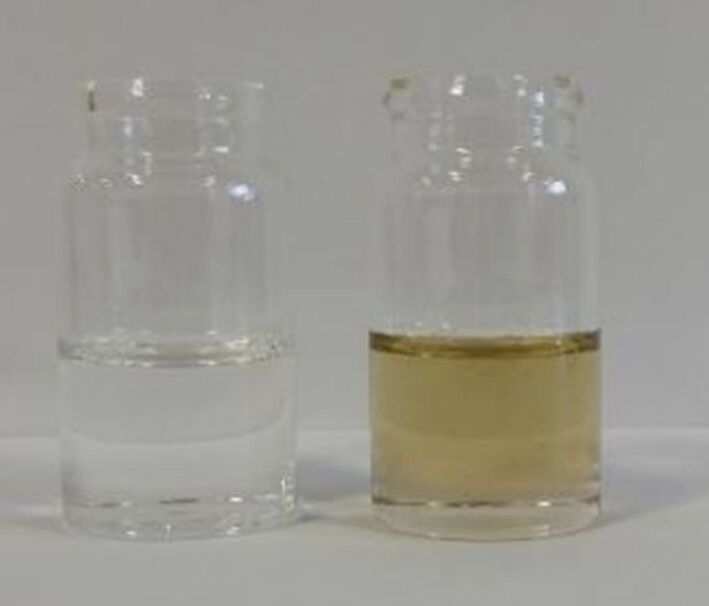
Comparison of choline chloride/D-fructose DES prepared via twin screw extrusion (left) and conventional heating (right). Taken from [44].

TSE overcame the problems identified by batch heating. The residence time for the continuous extrusion of the DES components was determined to be 4–8 minutes on average, producing quantities of ca. 0.4 kg h^−1^ (value of Reline 200 collected per hour) of DES [44]. The reaction times in the formation of DESs was greatly decreased, and as a result, thermal degradation was avoided in those DESs containing D-fructose due to the short exposure times to heat (Figure 5). The authors made a direct comparison of the determined space time yields (STY) for the batch preparation versus continuous preparation, which were significantly different. The STY determined for the extrusion process was four orders of magnitude greater at 3250,000 kg m^−3^ d^−1^, whereas batch synthesis was determined to be 500 kg m^−3^ d^−1^ [44]. Furthermore, these materials (particularly choline chloride/zinc chloride (1:2)) are known to be incredibly viscous and so very difficult to transport from the batch mixer into storage containers, but extrusion has avoided this issue as well, the material can be extruded directly into a storage container. This rules out the need for transfer and eliminates the loss of material upon that transfer. Therefore, it can be concluded that the use of TSE has improved the preparation of DESs and the quality of material obtained, which may in turn make them a more accessible media for metal processing or an alternative green solvent for synthesis [44].

#### Metal-organic frameworks (MOFs)

The above examples of cocrystal and DES formation describe systems which involve the formation of eutectic and intermolecular interactions upon mixing, but these did not involve the formation of a covalent bond. However, James et al. report on the formation of covalent bonds in metal-organic frameworks (MOFs) and discrete metal complexes by TSE, under solvent-free conditions or in the presence of stoichiometric amounts of MeOH [2]. There is a lot of commercial interest into the use of MOFs for gas capture and chemical separations [46]. Recently, the first commercial use of MOFs has been reported and this involves the adsorption of ethylene gas from the ripening of fruit and vegetables postharvest [47]. As the commercial interest and usage of MOFs increases, the manufacture of these materials, which typically require solvothermal techniques, has become a key research area.

**Scheme 6:**
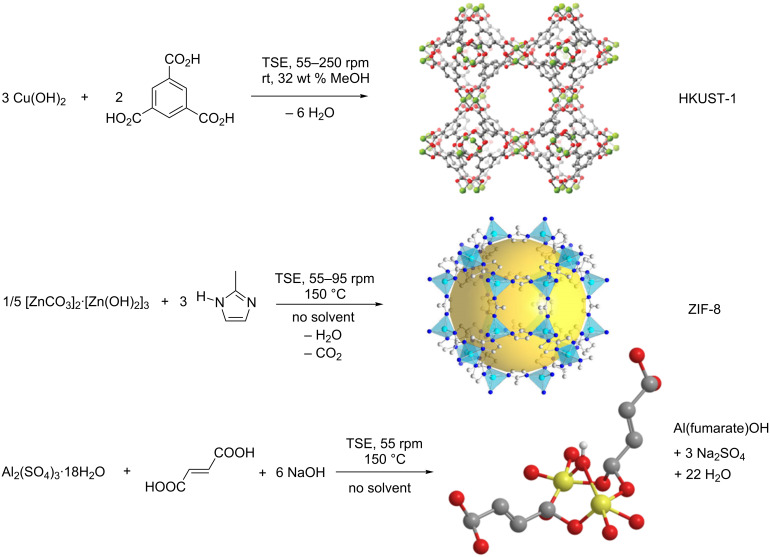
Synthesis of HKUST-1, ZIF-8 and Al(fumarate)OH by twin screw extrusion. Adapted from [2].

Mechanochemical synthesis of several MOFs has been reported typically by ball milling [48], and James et al. have scaled up the synthesis of HKUST-1, ZIF-8 and Al(fumarate)OH by TSE. Each synthesis involves the reaction between an organic ligand and metal salt. In the synthesis of ZIF-8 and Al(fumarate)OH, high temperatures were required in the absence of solvent, whereas the synthesis of HKUST-1 required stoichiometric amounts of EtOH at room temperature. STYs of 144,000 kg m^−3^ d^−1^ were reported for ZIF-8 and HKUST-1, and for the latter, the STY was three orders of magnitude greater than that reported for the conventional batch synthesis in the literature (Scheme 6) [2].

As with most examples discussed herein, the reaction times to form these MOFs were dramatically reduced from days (via solvothermal methods), to minutes (by TSE) [49]. Furthermore, the TSE products were of very high quality, comparable to the products obtained by batch, solvothermal methods. The N_2_ BET surface areas of extruded MOFs were similar to, or greater than, that of MOFs prepared in batch. PXRD analysis also indicated that highly crystalline materials were produced from the extrusion process, prior to any post process purification [2].

**Figure 6:**
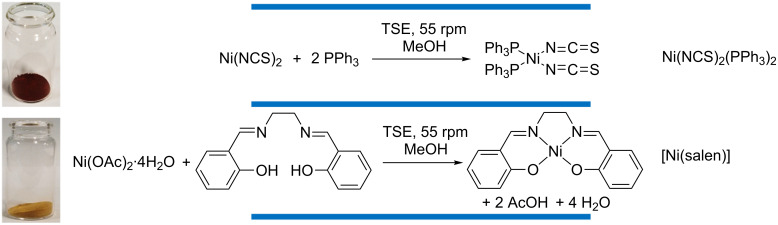
Synthesis of Ni(NCS)_2_(PPh_3_)_2_ and [Ni(salen)] by twin screw extrusion. Adapted from [2].

Two discrete metal complexes have been synthesised by extrusion, involving the reaction between salenH_2_ and nickel acetate dihydrate as well as the reaction between triphenylphosphine and nickel thiocyanate, both in the presence of stoichiometric amounts of MeOH (Figure 6) [2]. High-quality products were obtained, as determined by ^1^H NMR spectroscopy, PXRD analysis (which gave sharp diffraction patterns, indicating high crystallinity) and elemental analysis. James et al. report that both of these complexes were isolated and characterised with the only post process workup involved was heating in an oven for two hours [2], which is highly advantageous. Typically, workup of these complexes would involve isolating a precipitate through filtration, followed by drying to remove the copious amounts of MeOH employed as the reaction media.

## Conclusion

Organic synthesis is typically quite labour intensive and therefore industrialists are actively seeking ways to minimise the amount of labour required to manufacture organic compounds, particularly in an automated continuous fashion. In addition, they are also looking for techniques that still allow for compounds requiring many synthetic steps to be manufactured and preferably at a lower cost.

As discussed, extrusion has many roles within the food, polymer and pharmaceutical industries. Here we discussed how organic transformations have already been carried out by extrusion, hopefully allowing readers to understand that this technique could have a future in organic synthesis. To validate this, we have reviewed briefly how the technique has been used for inorganic synthesis and the preparation of cocrystals. This is the first time that the work reviewed here has been highlighted as a form of organic synthesis. In fact, the authors of the work included put a great emphasis on the processing and applications of the polymers and thus do not discuss any of the chemistry that is involved. This may be the reason why these transformations have been overlooked as organic synthesis, and it is hoped that we have highlighted this here.

Employing extrusion for chemical processes brings with it many advantages as discussed. However, there still remains some limitations that inhibits the potential of this technique in chemical synthesis, for example, reactions between two or more liquids have not been studied by extrusion and may be more difficult to carry out. In fact, there are still very few examples of this reported in ball mill reactions. Secondly, although it has been described that pyrophoric materials can be used in the extruder, through the use of *s*-Buli by Höcker et al., reagents that are potentially explosive or can be ignited when dry or exposed to friction are too hazardous to be used in an extrusion process, therefore chemistry involving azides or hydrazines for example, would need to be avoided.

In summary, extrusion is a technique that has great potential for use in organic synthesis. It has already been demonstrated as a method to scale up synthesis carried out by ball milling, therefore there is very little preventing its use for the organic reactions that have been reported to be successful by ball milling also. Condensation reactions (e.g., Knoevenagel condensations, Michael additions and Aldol reactions) in particular are the most obvious reaction to be successful by extrusion due to its general success in the ball mill, and as their reactions can be accelerated by the simple removal of water (by heating for example).

Extrusion provides a way to achieve intimate mixing of the reagents, it also allows for the extent of mixing to be fine-tuned (via modification of the screw configuration), the extruder itself can be heated to several hundred degrees and if required, small amounts of solvent can be added to accelerate reactions (liquid-assisted grinding). Therefore, it can be concluded that the extruder provides most, if not all of the parameters that conventional solvent-based synthesis can provide. In fact, in regards to the current drive towards a more sustainable environment, the extruder is advantageous as the amount of solvent required is either reduced or eliminated. Furthermore, typically the reaction times are greatly reduced and telescoping can be achieved in the extrusion process as discussed.
